# Inhibition of UCH-L1 in oligodendroglial cells results in microtubule stabilization and prevents α-synuclein aggregate formation by activating the autophagic pathway: implications for multiple system atrophy

**DOI:** 10.3389/fncel.2015.00163

**Published:** 2015-05-05

**Authors:** Katharina Pukaß, Christiane Richter-Landsberg

**Affiliations:** Department of Neuroscience, Molecular Neurobiology, University of OldenburgOldenburg, Germany

**Keywords:** glial cells, deubiquitinating enzymes, tubulin tyrosination, GFP-LC3, glial cell inclusions

## Abstract

α-Synuclein (α-syn) positive glial cytoplasmic inclusions (GCI) originating in oligodendrocytes (ODC) are a characteristic hallmark in multiple system atrophy (MSA). Their occurrence may be linked to a failure of the ubiquitin proteasome system (UPS) or the autophagic pathway. For proteasomal degradation, proteins need to be covalently modified by ubiquitin, and deubiquitinated by deubiquitinating enzymes (DUBs) before proteolytic degradation is performed. The DUB ubiquitin carboxyl-terminal hydrolase L1 (UCH-L1) is a component of the UPS, it is abundantly expressed in neuronal brain cells and has been connected to Parkinson’s disease (PD). It interacts with α-syn and tubulin. The present study was undertaken to investigate whether UCH-L1 is a constituent of ODC, the myelin forming cells of the CNS, and is associated with GCIs in MSA. Furthermore, LDN-57444 (LDN), a specific UCH-L1 inhibitor, was used to analyze its effects on cell morphology, microtubule (MT) organization and the proteolytic degradation system. Towards this an oligodendroglial cell line (OLN cells), stably transfected with α-syn or with α-syn and GFP-LC3, to monitor the autophagic flux, was used. The data show that UCH-L1 is expressed in ODC derived from the brains of newborn rats and colocalizes with α-syn in GCIs of MSA brain sections. LDN treatment had a direct impact on the MT network by affecting tubulin posttranslational modifications, i.e., acetylation and tyrosination. An increase in α-tubulin detyrosination was observed and detyrosinated MT were abundantly recruited to the cellular extensions. Furthermore, small α-syn aggregates, which are constitutively expressed in OLN cells overexpressing α-syn, were abolished, and LDN caused the upregulation of the autophagic pathway. Our data add to the knowledge that the UPS and the autophagy-lysosomal pathway are tightly balanced, and that UCH-L1 and its regulation may play a role in neurodegenerative diseases with oligodendroglia pathology.

## Introduction

A common pathogenic event in various neurodegenerative diseases is the failure to clear misfolded or aggregated proteins, which occur as protein inclusions in nerve cells or glia (Dohm et al., 2008; Jellinger, 2012). The inclusions often contain cytoskeletal proteins, a variety of heat shock proteins and ubiquitin, and are characteristic for each type of disease in composition, shape and size. α-Synuclein (α-syn) is prominently present in Parkinson’s disease (PD), dementia with Lewy bodies (DLB) and multiple system atrophy (MSA), a progressive adult-onset neurodegenerative disease with autonomic failure, symptoms of parkinsonism and ataxia (Jellinger and Lantos, 2010; Fernagut et al., 2014). α-Syn positive glial cytoplasmic inclusions (GCIs) originating in oligodendrocytes (ODC) are the characteristic hallmarks for the neuropathological diagnosis of MSA. The causes of α-syn overexpression and aggregation in ODC are not yet clear, however, impairments of the proteolytic degradation systems have been implicated to contribute to pathogenic consequences (Nixon, 2006; Xilouri and Stefanis, 2011; Richter-Landsberg and Leyk, 2013). α-Syn can be degraded either by the ubiquitin proteasome system (UPS) or by macroautophagy, hereafter referred to as autophagy (Webb et al., 2003; Lee et al., 2004; Vogiatzi et al., 2008; Riedel et al., 2010; Xilouri et al., 2013). The UPS and autophagy are closely working together and their activities need to be carefully balanced. Autophagy may act as a compensatory mechanism when the UPS is inhibited (Pandey et al., 2007; Schwarz et al., 2012; Richter-Landsberg and Leyk, 2013).

Autophagy is the only pathway by which large protein aggregates and organelles can be disposed of. It involves the formation and elongation of an isolation membrane, which sequesters cytoplasmic contents, resulting in an organelle surrounded by a double membrane, the autophagosome. After autophagosome fusion with the lysosome, the autolysosome is formed, where the contents are degraded by lysosomal enzymes (Nixon, 2006; Lamark and Johansen, 2012; Richter-Landsberg and Leyk, 2013). Autophagosome formation occurs throughout the cytoplasm, but to fuse with the lysosome, autophagosomes need to traffic toward the perinuclear region, a process requiring intact microtubules (MT) and dynein (Webb et al., 2004; Köchl et al., 2006). For proteasomal degradation, irreversibly damaged proteins are covalently modified by ubiquitin. This process involves a multistep procedure carried out by three different enzymes, namely E1 (ubiquitin-activating enzyme), E2 (ubiquitin-conjugating enzyme) and E3 (ubiquitin ligases) (Schwartz and Ciechanover, 2009; Huang and Figueiredo-Pereira, 2010). Substrates can be modified by conjugation to a single ubiquitin molecule or a polyubiquitin chain. K48-linked polyubiquitin chains generally target proteins for proteasomal degradation, while K63-linked protein chains are involved in various cellular processes. These include autophagy, cell sorting, and DNA repair processes (Todi and Paulson, 2011). The polyubiquitin chain attached to the protein needs to be removed by proteolysis before the protein is degraded by the proteasome. This is achieved by deubiquitinating enzymes (DUBs). DUBs comprise a large family of enzymes opposing the function of E3 ligases and are important regulators of the ubiquitin system (Amerik and Hochstrasser, 2004; Komander et al., 2009; Reyes-Turcu et al., 2009). They prevent ubiquitin degradation in conjunction with the substrate thereby maintaining ubiquitin homeostasis. Furthermore, they are critically involved in regulating protein stability and can modulate kinase cascades of signal transduction pathways. Their overexpression or impairment may contribute to neurodegenerative diseases and to the regulation of cell death and survival (Todi and Paulson, 2011).

We have recently shown that treatment of oligodendroglial cells with the broad range DUB inhibitor PR-619 caused the accumulation of ubiquitinated proteins and the formation of ubiquitin-positive protein aggregates, which colocalize with heat shock proteins, such as HSP70 and αB-Crystallin (Seiberlich et al., 2012). PR-619 did not affect proteasomal activity directly, but chronic inhibition of DUBs led to an overload of ubiquitinated proteins, which exhausted the UPS and autophagy was activated possibly as a compensatory mechanism (Seiberlich et al., 2013). Aggresome like structures were formed around the MT organizing center and DUB inhibition leads to MT stabilization similarly to the effects of taxol (Seiberlich et al., 2012).

Since PR-619 is a pan-inhibitor of DUBs, the question remained whether specific DUBs exert similar physiological responses and are involved in oligodendroglial pathology. In this respect the ubiquitin editing enzyme ubiquitin carboxyl-terminal hydrolase L1 (UCH-L1) is of interest. UCH-L1 is abundantly expressed in the brain and so far only has been examined and identified as a neuronal constituent. It localizes to synaptic vesicles and co-immunoprecipitates with α-syn (Liu et al., 2002; Amerik and Hochstrasser, 2004; Cartier et al., 2012), however, its precise functions remain unclear. Mutations in the UCH-L1 gene have been connected to PD and AD, and UCH-L1 has been found in Lewy bodies of PD and neurofibrillary tangles in patients with AD (Lowe et al., 1990; Yasuda et al., 2009). UCH-L1 directly interacts with α-tubulin (Kabuta et al., 2008), and overexpression of UCH-L1 was demonstrated to reduce MT formation *in vitro* (Bheda et al., 2010).

As mentioned above, ubiquitin-conjugated proteins also accumulate in neurodegenerative disorders with glial pathology, and MSA belongs to the group of synucleinopathies and has features of Parkinsonism (Jellinger and Lantos, 2010). ODC express α-syn, which aggregates under stressful conditions such as oxidative stress and proteasomal inhibition (Richter-Landsberg et al., 2000; Riedel et al., 2009; Pukass and Richter-Landsberg, 2014). ODC are dependent on an intact MT network, which is involved in transport processes and protein aggregate formation (Bauer et al., 2009). The present study was undertaken to investigate whether UCH-L1 is a constituent of ODC and associates with GCIs in MSA, and whether its pharmacological inhibition by LDN-57444 (LDN) affects cell morphology, MT formation and the proteolytic degradation system.

## Materials and Methods

### Ethics Statement

The care and treatments of animals were in accordance with the institutional guidelines for animal welfare of the University of Oldenburg, following the standards described by the German animal protection law (Tierschutzgesetz). The mere killing of rats for tissue removal is registered with the local authorities (Niedersächsisches Landesamt für Verbraucherschutz und Lebensmittelsicherheit) and reported on a regular basis as demanded by law but needs no further approval if no other treatment is applied before killing.

### Study Subjects

Tissue samples from MSA- and from PD-cases were obtained from the Department of Neuropathology, Klinikum Bremen-Mitte, Germany. They were diagnosed during the period from 1974 to 2006. In this study, we analyzed pontine sections of two patients with MSA, one patient with PD, and one patient with an astrocytoma as a control. Brain tissue was fixed in 10% formalin at time of autopsy, cut into tissue blocks, and processed in paraffin wax using standard protocols. Tissue blocks were cut into 3 μm thick sections.

### Materials and Antibodies

Cell culture media were from Gibco/BRL (Grand Island, NY, USA). Poly-L-lysine (PLL) and neutral red (NR) were purchased from Sigma-Aldrich (Munich, Germany). LDN was from LifeSensors (Philadelphia, PA, USA). Bafilomycin A1 (Bf) was purchased from Merck Millipore (Darmstadt, Germany). For Western blot analysis the following antibodies were used, the working dilutions are given in brackets: anti α-tubulin mouse monoclonal antibody (mAb) (1:1,000) and mouse mAb anti acetylated α-tubulin (1:1,000) were from Sigma-Aldrich (Munich, Germany). Rabbit polyclonal antibody (pAb) anti detyrosinated α-tubulin (1:1,000) was from Merck Millipore (Darmstadt, Germany) and rat mAb anti tyrosinated α-tubulin clone YL1/2 (1:1,000) was from Santa Cruz (Dallas, TX, USA). Rabbit pAb anti LC3 (1:500) and rabbit pAb PGP 9.5 against UCH-L1 (1:1,000) were from abcam (Cambridge, UK). Rabbit pAb anti green fluorescent protein (GFP) (1:1,000) was from Invitrogen (Grand Island, NY, USA). Mouse mAb anti Beclin-1 (1:200) was from nanoTools (Teningen, Germany). SNL-4, a rabbit pAb made against a synthetic peptide corresponding to residues 2–12 in human α-syn, was from Dr. Virginia Lee (Philadelphia, PA, USA). Rabbit pAb anti-myelin basic protein (MBP) (1:1,000) was a generous gift from Dr. Jean-Marie Matthieu (University Lausanne, Switzerland). HRP-conjugated anti-mouse IgG (1:10,000) and anti-rabbit IgG (1:10,000) were from Jackson ImmunoResearch (West Grove, PA, USA).

For immunohistochemical studies, the following antibodies were used: Mouse mAb LB509 (1:500) directed against α-syn was from Invitrogen (Grand Island, NY, USA). Rabbit pAb PGP 9.5 against UCH-L1 (1:100) was from abcam (Cambridge, UK).

### Immunohistochemistry

For DAB-staining antigen retrieval was done using Vector unmasking solution (Vector Laboratories, Burlingame, CA, USA) according to the manufacturer’s instructions. Endogenous peroxidase activity was quenched using 1% H_2_O_2_ and 50% methanol in phosphate-buffered saline (PBS). Sections were blocked in 3% bovine serum albumin (BSA) in PBS supplemented with 0.2% Triton-X 100 for 1 h at room temperature. Sections were incubated with primary antibodies mouse mAb LB509 and rabbit pAb PGP 9.5 diluted in the blocking solution overnight at 4°C. After washing, sections were incubated with biotinylated secondary antibodies (1:100) diluted in blocking solution for 2 h at room temperature. After washing, sections were incubated with the Vectastain ABC-complex (Biologo, Kronshagen, Germany) for 1 h at room temperature. Bound antibodies were visualized by incubation with DAB-solution for 5 min. Nuclei were stained with hematoxylin for 5 min. After washing with water, sections were dehydrized and mounted with D.P.X. (Sigma-Aldrich, Munich, Germany).

Double immunolabeling was carried out after antigen retrieval using Vector unmasking solution (Vector Laboratories, Burlingame, CA, USA) according to the manufacturer’s instructions. Sections were blocked in 3% BSA in PBS supplemented with 0.2% Triton-X 100 for 1 h at room temperature. First antibodies (LB509 and PGP 9.5) were diluted in blocking solution and incubated at 4°C overnight. After three washes for 30 min with 0.1 M Tris, pH 7.6, sections were incubated with Dylight 594-conjugated goat anti-mouse (1:500) and Dylight 488-conjugated goat anti-rabbit (1:500) secondary antibodies (Thermo Scientific, Rockford, IL, USA) and with 0.1 μg/ml 4’6-diamidino-2-phenylindole (DAPI; Sigma-Aldrich, Munich, Germany) diluted in blocking solution for at least 3 h at room temperature. Autofluorescence was blocked using a 0.3% Sudan Black B solution in 70% ethanol (Romijn et al., 1999). Sections were mounted with Mowiol® 4–88 (Sigma-Aldrich, Munich, Germany).

DAB-staining was analyzed using an Olympus microscope IX70 with bright-field light sources equipped with a digital camera. Double labeled fluorescence sections were studied using a Zeiss epifluorescence microscope (Oberkochen, Germany) equipped with a digital camera using a plan-neofluar objective (×100) or a Leica TCS SL confocal laser scanning microscope (Wetzlar, Germany).

### Cell Culture

In this study OLN-93 cells (Richter-Landsberg and Heinrich, 1996), OLN-93 cells stably transfected with the longest tau isoform and with the wild-type human α-syn, named OLN-t40-α-syn cells (Riedel et al., 2009), and OLN-93 cells stably transfected with the longest tau isoform and with the A53T mutant human α-syn, named OLN-t40-A53T (Riedel et al., 2009), were used. Additionally, a newly established cell line, OLN-t40-α-syn cells stably transfected with GFP-LC3, was used to monitor the influence of the inhibition of UCH-L1 activity on the autophagic flux. OLN-t40-α-syn cells were transfected with GFP-LC3 plasmid (InvivoGen, Toulouse, France) containing the Zeocin resistance gene by using lipofection with Metafectene® Pro (Biontex, Martiensried, Germany). After selection in Dulbecco’s modified Eagle medium (DMEM) containing 600 μg/ml Zeocin, the cells were screened for tau, α-syn, and for GFP-LC3 expression by western blot and indirect immunofluorescence. A stable cell line was established designated OLN-t40-α-syn-GFP-LC3 cells. Cells were kept in DMEM supplemented with 10% heat-inactivated fetal bovine serum (FBS), 2 mM Glutamine, 50 U/ml penicillin (P), and 50 μg/ml streptomycin (S) at 37°C and 10% CO_2_ (Richter-Landsberg and Heinrich, 1996).

ODC were prepared as previously described (Goldbaum and Richter-Landsberg, 2004). Briefly, primary cultures of glial cells were prepared from the brains of newborn Wistar rats sacrificed by decapitation, and ODC were mechanically removed after 10–14 days in culture by shaking the flasks on an orbital lab-shaker (New Brunswick Scientific, Edison, NJ, USA). ODC precursor cells were replated on PLL-coated culture dishes (1.2 × 10^6^ cells/6 cm dish) supplemented with glass cover slips (Fisher Scientific, Schwerte, Germany). Cells were grown in serum free DMEM supplemented with 2 mM Glutamine, 50 U/ml penicillin, 50 μg/ml streptomycin, 5 μg/ml insulin, 5 μg/ml transferrin, and 5 ng/ml sodium selenite (Roche Diagnostics, Mannheim, Germany) in a 10% CO_2_ atmosphere for up to 10 days. After 5 days these cultures contain a highly enriched population of differentiated ODC with a mature morphology.

### Neutral Red Assay

To assess the cytotoxic potential of LDN on oligodendroglial cells, OLN-t40-α-syn cells were plated on PLL-coated 96-microwell cell culture plates at a density of 3,000 cells per well. The cells were incubated in DMEM/10% FBS for 24 h. Thereafter the growth medium was removed and fresh medium (100 μl/well) was added with LDN at the indicated concentrations and cells were incubated for 18 h. Cells were then washed with PBS and incubated for 4 h in medium containing NR (0.005%) (100 μl/well). Damaged or dead cells lose the ability to retain NR. Cells were washed with PBS and NR was extracted with a solution of acetic acid (1%) and ethanol (50%) (100 μl/well). The plate was agitated for 2 h and the absorbance of the extracted dye was determined in a SpectraCount® Microplate Photometer at 540 nm. Data are expressed as percentage of the untreated control cells and values represent the means ± SD of 16 microwells each of at least two independent experiments (*n* = 32).

### Immunoblot Analysis

For the detection of UCH-L1 in human (HB) and rat brain (RB) 0.1 g of brain tissue were homogenized in 1 ml 1 × RIPA (20 mM Tris/HCl (pH 7.5), 150 mM NaCl, 1 mM EGTA, 1 mM Na_2_-EDTA, 1% NP-40, 1% sodium deoxycholate, and a mixture of protease inhibitors (Mini Complete; Roche Diagnostics, Mannheim, Germany)) by sonification. Homogenates were supplemented with sample buffer containing 5% SDS and boiled for 10 min. Dorsal root ganglion (DRG) neurons were cultured in DMEM 10% FBS + P/S + NGF + B27 supplement for 14 days until they achieved the neuronal phenotype. N2A cells and astrocytes (AST) were cultured for 3 days or 2 weeks, respectively, in DMEM/10% FBS.

Cellular monolayers of control and treated cells were washed with PBS once, scraped off in sample buffer containing 1% SDS, and boiled for 10 min. Protein contents were determined according to Neuhoff et al. (1979). For immunoblotting total cellular extracts (10–30 μg protein per lane) were separated by one-dimensional SDS-polyacrylamide gel electrophoresis (SDS-PAGE) using 8.75–11.25% polyacrylamide gels and transferred to nitrocellulose membranes (Whatman, Dassel, Germany; 0, 2 μm) or to PVDF membranes (BioRad, Hercule, CA, USA) for the detection of LC3. The blots were saturated with Tris-buffered saline (TBS) (20 mM Tris, 136.8 mM NaCl, pH 7.5) containing 5% dry milk and incubated with the individual antibodies overnight at 4°C. After washing with TBS with 0.1% v/v Tween 20 (TBS-T), incubation with HRP-conjugated anti-mouse (1:10,000) or anti-rabbit (1:10,000) antibody was carried out for 1 h at RT. After washing with TBS-T, blots were visualized by the enhanced chemiluminescence (ECL) procedure as described by the manufacturer (Thermo Scientific, Rockford, IL, USA). All experiments were carried out at least three times with similar results. Quantitative evaluations of the immunoblots were carried out by densitometric scanning and ImageQuant software (Molecular Dynamics, Sunnyvale, CA, USA).

### Immunocytochemistry

Naive OLN-93 cells and OLN-t40-α-syn cells (1.2 × 10^5^ cells/10 cm dish), and OLN-t40-α-syn-GFP-LC3 cells (1.6 × 10^5^ cells/10 cm dish) were cultured on PLL-coated glass coverslips for 72 h in DMEM/10% FBS and P/S and then subjected to treatment as indicated. Primary ODC (1.2 × 10^6^ cells/6 cm dish) were cultured for 3 h up to 10 days on PLL-coated glass coverslips in DMEM without any treatment for investigating the developmental expression of UCH-L1. After washing with PBS, cells were fixed and permeabilized with ice-cold methanol for 7 min or fixed with 3% paraformaldehyde for 15 min and permeabilized with 0.1% Triton-X 100 for 20 min for incubating with the anti-α-syn antibody SNL-4. Cells were washed three times with PBS and incubated for 30 min with 5% BSA in PBS for blocking unspecific binding sites. Cells were incubated overnight at 4°C with the following antibodies (the working dilutions are given in brackets): rabbit pAb anti-α-syn (SNL-4; 1:500), mouse mAb anti-α-tubulin (1:250), mouse mAb anti-acetylated α-tubulin (1:250), rat mAb anti-tyrosinated α-tubulin clone YL1/2 (1:200), rabbit pAb anti-detyrosinated α-tubulin (Glu-tubulin) (1:200), rabbit pAb PGP 9.5 against UCH-L1 (1:100), mouse mAb against the chondroitin sulfate proteoglycan NG2 (Millipore, Billerica, MA, USA; 1:200) as a marker for oligodendrocyte precursor cells, mouse mAb anti-2′, 3′-cyclic nucleotide 3′-phosphodiesterase (CNP; Sigma, Munich, Germany; 1:250) as a marker for mature ODC. After washing with PBS, cells were incubated for 1 h with Dylight 594-conjugated (1:500) goat secondary antibodies (Thermo Scientific, Rockford, IL, USA), Dylight 488-conjugated (1:500) goat secondary antibodies (Thermo Scientific, Rockford, IL, USA), or fluorescein isothiocyanate-conjugated (1:100) donkey anti rat antibody (Jackson ImmunoResearch, West Grove, PA, USA), washed with PBS, and mounted. Nuclei were stained with 4′, 6-diamidino-2-phenylindole (DAPI; 1.5 μg/ml) included in the mounting medium (Vectashield, Vector Laboratories, Burlingame, CA, USA).

For gated Stimulated Emission Depletion (STED) laser scanning microscopy the Abberior STAR 488-conjugated (1:100) goat anti mouse secondary antibody (Abberior, Göttingen, Germany) was used and the cells were mounted with Mowiol® 4–88 (Sigma-Aldrich, Munich, Germany).

Fluorescent labeling was studied using a Zeiss epifluorescence microscope (Oberkochen, Germany) equipped with a digital camera using a plan-neofluar objective (×40, ×100). Additionally the newly established confocal laser scanning microscopy or gated STED laser scanning microscopy from Leica (Leica TCS SP5 X microscope (Wetzlar, Germany)) was used. Images were acquired with a 100× immersion objective (HCX PL APO 100.0× OIL STEDorange, NA = 1.4) using a white light laser (confocal laser) with a hybrid detector (HyD) or the STED laser. STED stacks were deconvolved with theoretical point spread functions using Huygens Essential deconvolution software (SVI, Hilversum, Netherlands). Confocal stacks and STED stacks were processed with ImageJ (NIH, Bethesda, MD, USA). All experiments were carried out at least three times with similar results.

### RNA Extraction and Reverse Transcription

RNA from ODC (2.7 × 10^6^ cells) was isolated with RNeasy (Quiagen, Hilden, Germany) as described by the manufacturer for animal cells. RNA was dissolved in diethylpyrocarbonate (DEPC)-treated water and quantified by spectrophotometry. About 1 μg of RNA was used for reverse transcription in a final volume of 25 μl. First-strand synthesis was performed with 0.5 mM of each dNTP, 0.5 μM each of oligo-dT_18_, and random hexamer primer, 5 μl of 5× M-MLV buffer, 25 U RNAsin, and 200 U M-MLV reverse transcriptase (Promega, Madison, WI, USA). After a denaturation step and incubation at 37°C for 1 h, the reaction mixture was diluted to 65 μl with DEPC-treated water and stored at −20°C. Subsequently, 1 μl was used for polymerase chain reaction analysis.

#### PCR and Primers

Primers were synthesized by Biomol (Hamburg, Germany): rat UCH-L1 5′-CCCTTGGTTTGCAGCTTTAG-3′ (nt 777–796) and 5′-CACATCCAAGGCCGTAACTT-3′ (nt 964–983) (Accession no.: NM_017237). For the analysis of the MBP gene following primers were used: 5′-GACCCTCACAGCGACACGGAT-3′ (nt 41–61) and 5′-CTGCTGAGGGACAGGCCTCTC-3′ (nt 336–356) (Roach et al., 1983).

Control experiments were carried out with the following primers for glyceraldehyde-3-phosphate dehydrogenase (GAPDH): 5′-CCCACGGCAAGTTCAACGGCA-3′ (nt 220–240) and 5′-TGGCAGGTTTCTCCAGGCGGC-3′ (nt 805–825) (Fort et al., 1985). PCR reactions were carried out as previously described (Goldbaum et al., 2008).

#### Statistics

Arbitrary units were determined by densitometric scanning of the individual protein bands, and set in relation to α-tubulin. Data are expressed as % of the control value, set as 100%. Results are expressed as mean ± SD from at least three independent experiments. Statistical evaluation was carried out by Student’s *t*-test: *p < 0.05 significant and ***p < 0.001 highly significant.

## Results

### The Deubiquitinase UCH-L1 is Abundant in ODC and Associates with GCIs in the Brains of Patients with MSA

**Figure 1:**
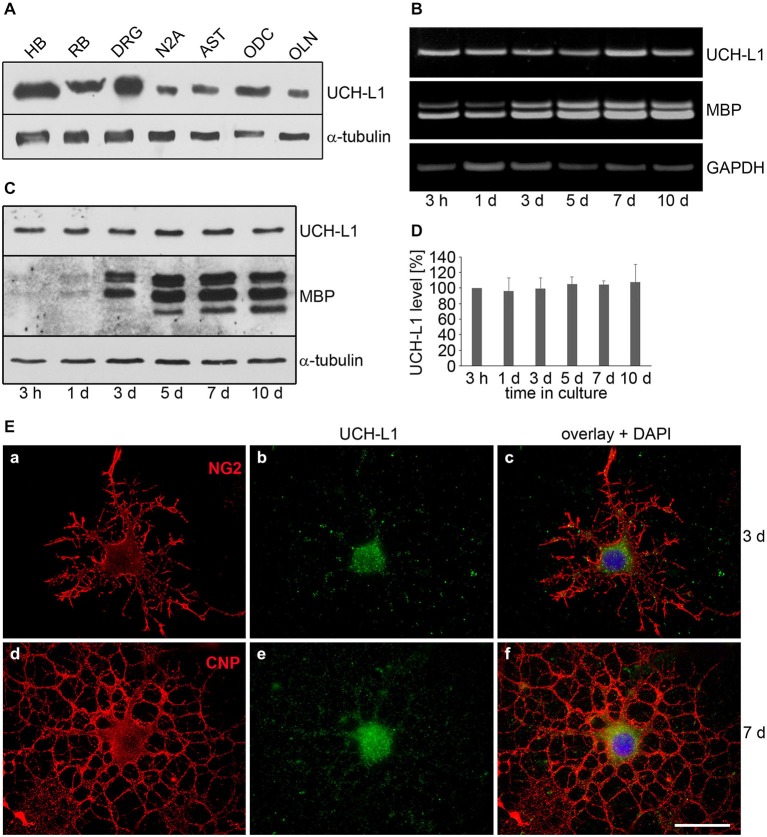
**The deubiquitinase UCH-L1 is expressed in rat brain oligodendrocytes (ODC). (A)** Immunoblot analysis. Cell extracts of human brain (HB), rat brain (RB), dorsal root ganglion neurons (DRG), N2A cells (N2A), astrocytes (AST), primary rat brain ODC, and OLN-93 cells (OLN) were subjected to immunoblot procedure using the antibodies as indicated on the right. **(B)** mRNA analysis. ODC were cultured for the indicated times. mRNA was extracted and subjected to RT-PCR. UCH-L1 was analyzed in relation to Glyceraldehyde-3-phosphate-Dehydrogenase (GAPDH) and Myelin basic protein (MBP) mRNA. The PCR-products representing the different mRNAs are marked on the right. **(C)** ODC were cultured for the indicated times. Cell extracts were subjected to immunoblot procedure using the antibodies as indicated on the right. **(D)** Quantitative evaluation of the blot shown in **(C)** was carried out using ImageQuant software. Data represent the mean ± SD from three independent experiments. UCH-L1 levels were normalized to α-tubulin and the total amount of the control was set to 100%. Statistical evaluation was carried out by Student’s *t*-test: the differences are not significant. **(E)** Indirect immunofluorescent staining. ODC were cultured for 3 or 7 days. Indirect immunofluorescence staining was carried out with antibodies against the chondroitin sulfate proteoglycan NG2 (a), 2′, 3′-cyclic nucleotide 3′-phosphodiesterase (CNP; d), and UCH-L1 (green). Nuclei were stained with DAPI (blue). Scale bar: 20 μm.

To investigate whether UCH-L1 is a constituent of ODC, primary cultures of RB ODC were extracted and subjected to immunoblot procedure, and compared to cellular extracts of HB, RB, DRG, AST neuronal N2A cells and the oligodendroglial cell line OLN-93 (OLN, see below). Figure 1A depicts that UCH-L1 is present in ODC and OLN-93 cells at a lower level than in DRG neurons. Furthermore RT-PCR analysis and immunoblot procedure were carried out to assess the regulation of UCH-L1 during culture maturation (Figures 1B,C). The data demonstrate that UCH-L1 mRNA is present in ODC, and that the protein level does not significantly change during *in vitro* differentiation for up to 10 days (Figure 1D), while the levels of MBP mRNA and protein increase during that time. Indirect immunofluorescence further demonstrates that UCH-L1 immunoreactivity is detected in the cell soma and throughout the cellular processes and has a punctuated appearance (Figure 1E).

**Figure 2:**
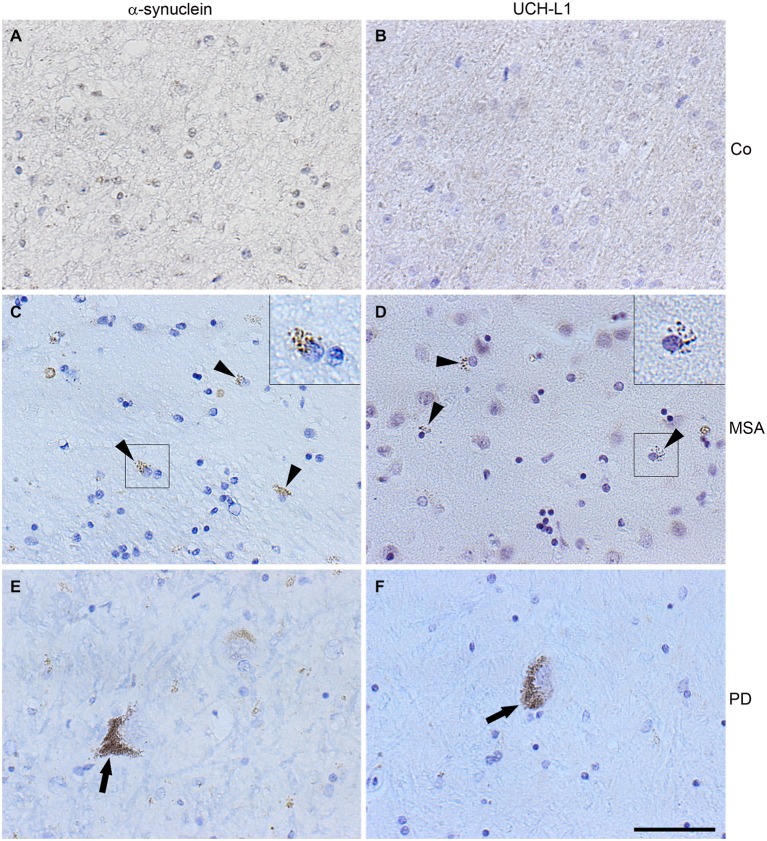
**α-Synuclein and UCH-L1 are present in Lewy bodies and glial cytoplasmic inclusions**. Immunohistochemistry of pontine sections using antibodies against α-syn (LB509) and UCH-L1, as indicated on the top, was carried out. The results reveal that glial cytoplasmic inclusions (arrow heads) and Lewy bodies (arrows), which do not occur in unaffected brain used as a control (Co) **(A,B)**, positively stain for α-syn **(C,E)** and UCH-L1 **(D,F)**. Scale bar: 50 μm.

**Figure 3:**
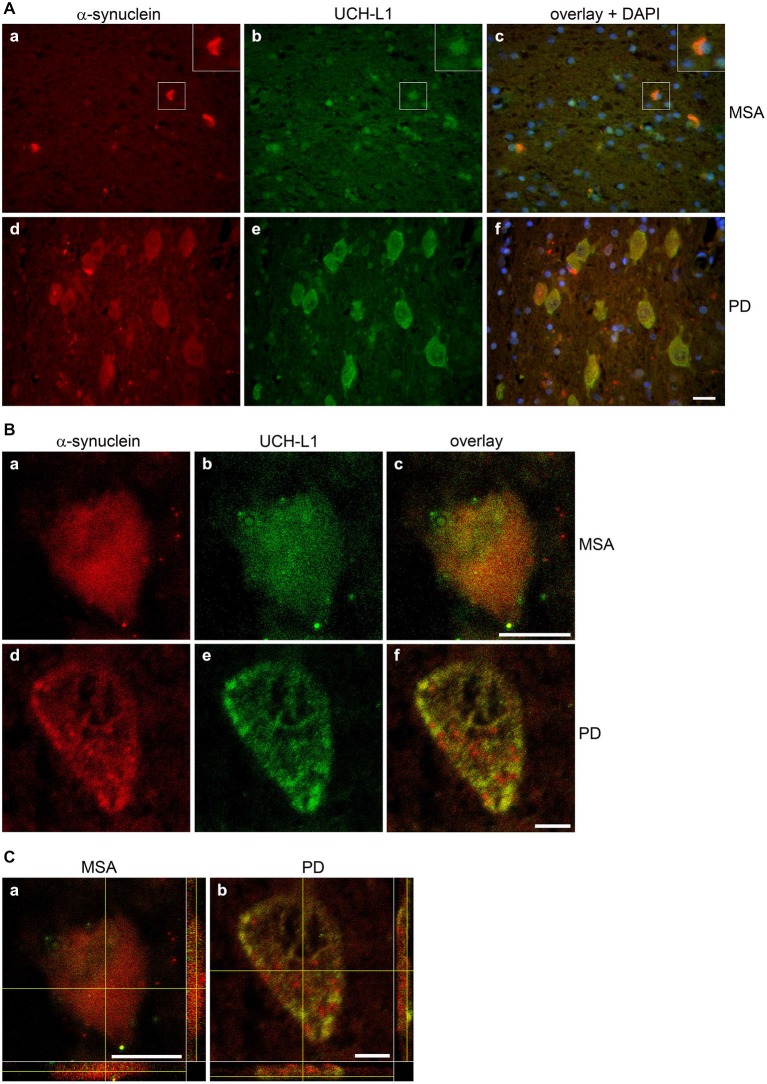
**Colocalization of α-synuclein and UCH-L1 in glial cytoplasmic inclusions and Lewy bodies. (A)** Double label immunohistochemistry of sections of multiple system atrophy (MSA) (a–c) and Parkinson’s disease (PD) brains (d–f) using antibodies against α-syn (LB509) and UCH-L1 (PGP 9.5), as indicated on the top. Overlay with DAPI (blue). Scale bar: 20 μm. **(B)** Confocal images (one section: 0.3 μm) of a glial cytoplasmic inclusion (a–c) and a Lewy body (d–f) at a higher magnification are shown. Scale bar: 5 μm. **(C)** Three dimensional reconstructions of confocal images (18 sections in a; 36 sections in b) corroborate that α-syn and UCH-L1 colocalize in GCIs and LBs (marked by a yellow cross). Reconstructed orthogonal projections are presented as viewed in the x-z (bottom) and y-z (right) planes. Scale bar: 5 μm.

To test the presence of UCH-L1 in MSA, immunohistochemistry of formalin-fixed, paraffin-embedded brain tissue sections of two MSA cases, one patient with PD, and one patient with an astrocytoma as a control with no GCIs was carried out. Pons tissue was chosen as a brain region undergoing extensive damage in MSA (Wenning et al., 2008). As opposed to the control (Figures 2A,B), GCIs occurred in both MSA cases and were immunostained with antibodies against α-syn and UCH-L1 (Figures 2C,D). Similarly, corroborating the finding of Lowe et al. (1990), UCH-L1 immunoreactivity was detectable in Lewy bodies in PD (Figure 2F). Furthermore a-syn was detectable in LBs in PD (Figure 2E). Double immunofluorescence labeling reveals that UCH-L1 colocalizes with α-syn in some but not all GCIs and in Lewy bodies (Figure 3A), which was further supported by confocal microscopy (Figure 3B). Orthogonal projections of confocal sections confirm these results (Figure 3C). Hence, UCH-L1 is a constituent of GCIs in cases of MSA and similarly as observed in Lewy bodies is colocalized with α-syn immunoreactivity.

### Inhibition of UCH-L1 by LDN Leads to MT Reorganization and Stabilization in Oligodendroglial Cells

**Figure 4:**
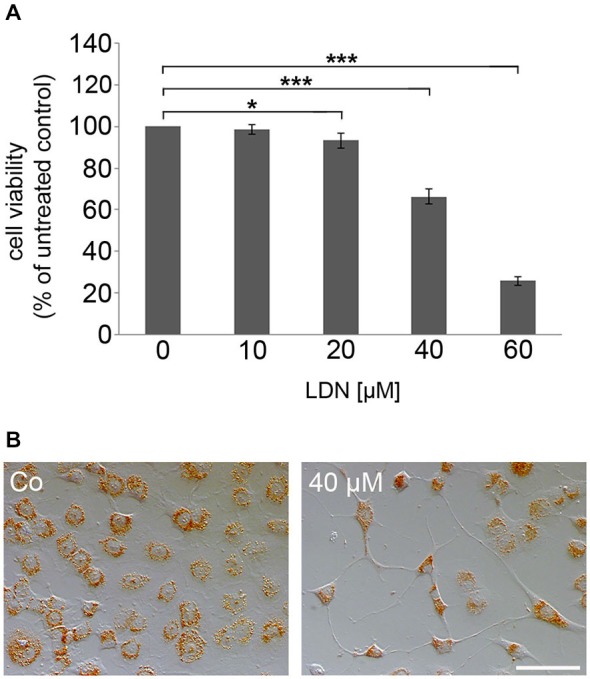
**The specific UCH-L1 inhibitor LDN causes concentration dependent cell death in oligodendroglial cells. (A)** Neutral red (NR) survival assay of OLN-t40-α-syn cells exposed to increasing concentrations of LDN. Cells were treated with 0–60 μM LDN for 18 h. Cell viability was determined by NR uptake. Data are expressed as percentage of untreated control cells. Values represent the means ± SD of 16 microwells of two independent experiments (*n* = 32). *p < 0.05 significant and ***p < 0.001 highly significant.** (B)** Cells were incubated with 40 μM LDN for 18 h (Co, untreated control). Thereafter cells were incubated with medium containing 0.005% NR and photographed. Scale bar: 100 μm.

To test whether inhibition of UCH-L1 has an influence on process outgrowth, MT organization and α-syn aggregate formation, the oligodendroglial cell line OLN-93 stably transfected with α-syn and the MT associated protein tau, namely OLN-t40-α-syn cells, was used (Richter-Landsberg and Heinrich, 1996; Riedel et al., 2009). These cells constitutively express small non-fibrillar α-syn aggregates. Cells were treated with LDN, which acts as a site-directed inhibitor of UCH-L1 inhibiting the hydrolase activity (Liu et al., 2003; Gong et al., 2006). To determine whether LDN exerts cytotoxic effects, cells were incubated with increasing concentrations of LDN (10–60 μM) for 18 h, and NR assay was carried out. Figure 4A depicts that LDN caused cytotoxic effects in a concentration dependent manner and that at 40 μM cell viability was determined as about 60%. This was sustained by microscopical inspection of the cells after NR uptake indicating that after treatment with LDN the amount of living cells capable of taking up NR was greatly impaired (Figure 4B). Furthermore cells appeared with long cellular processes after LDN (40 μM) incubation. For the following experiments this concentration was used and cells were incubated for 18 h.

**Figure 5:**
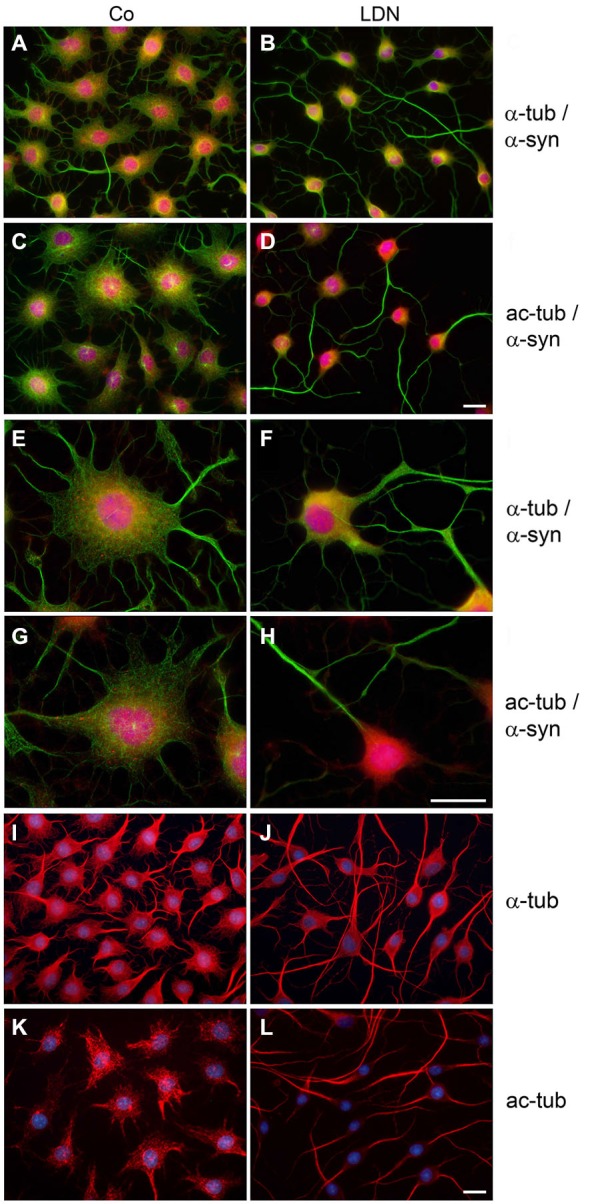
**LDN induces reorganization of microtubules and the clearance of α-synuclein puncta in oligodendroglial cells overexpressing α-synuclein**. OLN-t40-α-syn cells **(A–H)** or OLN-93 cells **(I–L)**, not expressing α-syn or tau, were incubated with 40 μM LDN for 18 h (Co, untreated control). OLN-t40-α-syn cells were subjected to indirect immunofluorescence staining with antibodies against α-syn (red) and α-tubulin (α-tub; green) or acetylated α-tubulin (ac-tub; green) (**A–H**, as indicated), OLN-93 cells with antibodies against α-tub (red) or ac-tub (red) as indicated. Nuclei were stained with DAPI (blue). Overlay images are shown. Scale bar: 20 μm.

To further assess the influence of LDN on process outgrowth and abundance of α-syn aggregates in these cells, indirect immunofluorescence staining with antibodies against α-tubulin and α-syn was carried out. Additionally, an antibody against acetylated α-tubulin (ac-tubulin) was used. Tubulin acetylation is considered to occur on stable MTs and to be an indicator of MT stability (Janke and Bulinski, 2011; Perdiz et al., 2011). In control cells MTs are distributed throughout the soma and cellular processes and were stained by antibodies against α-tubulin (Figures 5A,E) and against ac-tubulin (Figures 5C,G). Small α-syn positive aggregates are seen (Figures 5A,C,E,G), which are abolished after LDN treatment (Figures 5B,D,F,H). After inhibition of UCH-L1 activity cellular processes are elongated and ac-tubulin is mainly prominent within the processes and not apparent in the soma (Figures 5D,H). This is similarly observed in naive OLN-93 cells without endogenous α-syn and tau (Figures 5I–L), and also in OLN cells expressing the A53T α-syn mutation (data not shown).

**Figure 6:**
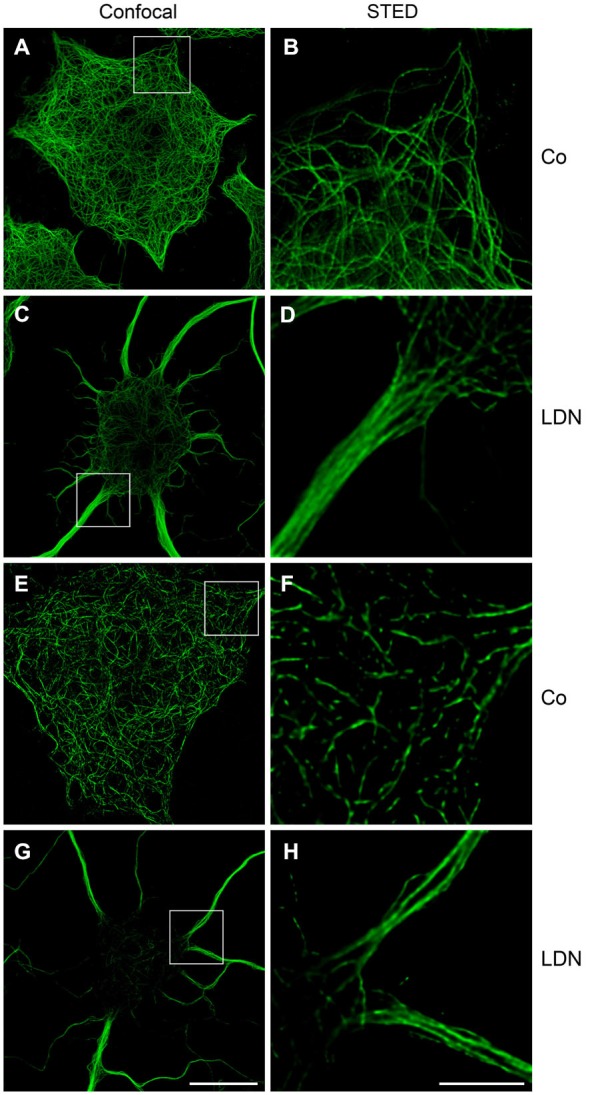
**Microtubule bundling and acetylation after LDN treatment: Confocal and STED microscopy**. OLN-t40-α-syn cells were incubated with 40 μM LDN for 18 h (Co, untreated control). Cells were subjected to indirect immunofluorescence staining with antibodies against α-tubulin **(A–D)** and acetylated α-tubulin **(E–H)**. Confocal images (one section: 0.21 μm) and STED images (one section: 0.13 μm) are shown. Scale bar confocal images **(G)**: 20 μm. Scale bar STED images **(H)**: 5 μm.

To monitor MT organization and the localization of ac-tubulin within the cellular extensions more closely, confocal microscopy and STED microscopy (Hein et al., 2008) was carried out. This shows that under control conditions MTs labeled with antibodies against α-tubulin are evenly distributed, while after LDN treatment MTs appear as thick bundles within the cellular extensions (Figures 6A–D). Figure 6F depicts that in untreated control cells ac-tubulin is prominent in the cell soma. However, STED microscopy demonstrates that MTs are not continuously acetylated, but rather smaller elongated patches are visible, which are interrupted by small gaps of non-acetylated tubulin (Figure 6E). After treatment with LDN ac-tubulin is translocated to the cellular processes and the thick MT bundles are intensely labeled by antibodies against ac-tubulin (Figures 6G,H). Ac-tubulin is continuously present along the MTs and not interrupted by gaps, as seen in untreated control cells. Hence LDN treatment leads to the recruitment of ac-tubulin to the cell processes, which may promote MT stabilization.

**Figure 7:**
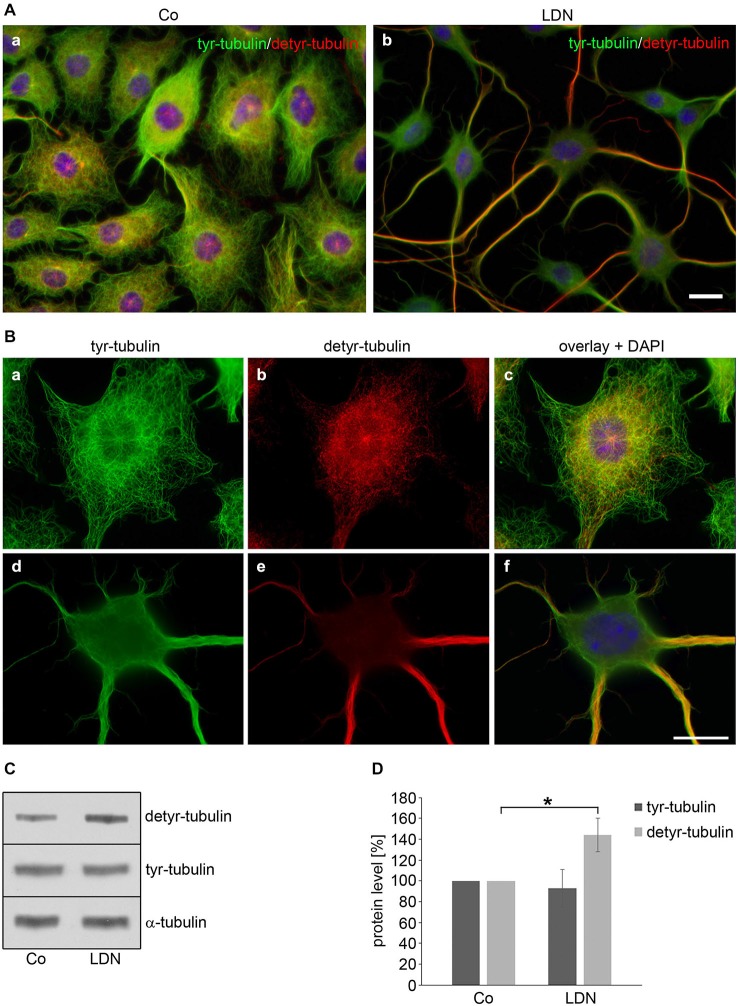
**Effects of LDN treatment on microtubule detyrosination. (A,B)** Indirect immunofluorescence staining: OLN-t40-α-syn cells were left untreated (**A**,a,**B**,a–c) or incubated with 40 μM LDN for 18 h (**A**,b,**B**,d–f). Cells were subjected to indirect immunofluorescence staining using antibodies against tyrosinated α-tubulin (tyr-tubulin; green) and detyrosinated tubulin (detyr-tubulin; red). Nuclei were stained with DAPI (blue). Scale bar: 20 μm. **(C)** Immunoblot analysis: OLN-t40-α-syn cells were incubated with 40 μM LDN for 18 h. Co, untreated control. Cell lysates were subjected to immunoblot analysis with the antibodies as indicated on the right. **(D)** Quantitative evaluation of the blot shown in **(C)** was carried out using ImageQuant software. Data represent the mean ± SD from three independent experiments. Tyr-tubulin and detyr-tubulin levels were normalized to α-tubulin and the total amount of the control was set to 100%. Statistical evaluation was carried out by Student’s *t*-test: *p < 0.05 significant.

Since tubulin acetylation is not strictly associated with stable MTs, we determined the effect of LDN on the tyrosination state of tubulin. Detyrosination of α-tubulin stabilizes MTs indirectly and is an indication of enhanced MT stability (Janke and Bulinski, 2011). Using antibodies against tyrosinated and detyrosinated α-tubulin, indirect immunofluorescence demonstrates that tyrosinated α-tubulin (tyr-tubulin) is distributed throughout the cell soma and processes in control cells, while detyrosinated α-tubulin (detyr-tubulin) is mainly expressed in the cell soma and not at the cellular boundaries and protrusions (Figures 7Aa,Ba–c). After LDN treatment detyr-tubulin is almost exclusively concentrated within the long cellular processes and associated with the thick bundles and hardly visible in the cell soma (Figures 7Ab,Bd–f). Furthermore, immunoblot analysis of cell extracts reveals that LDN leads to an increase of detyr-tubulin of about 44% (±16%) as compared to the untreated control (Figures 7C,D). In contrast thereto, the level of acetylated α-tubulin did not increase (Figure 9). Also, LDN had no influence on tau or tau phosphorylation (data not shown), and as mentioned above elongation of the cellular processes was independent of the presence of tau or α-syn and also seen in OLN-93 cells which do not contain both proteins (Figures 5I–L).

### Inhibition of UCH-L1 Leads to the Clearance of α-Synuclein Aggregates

**Figure 8:**
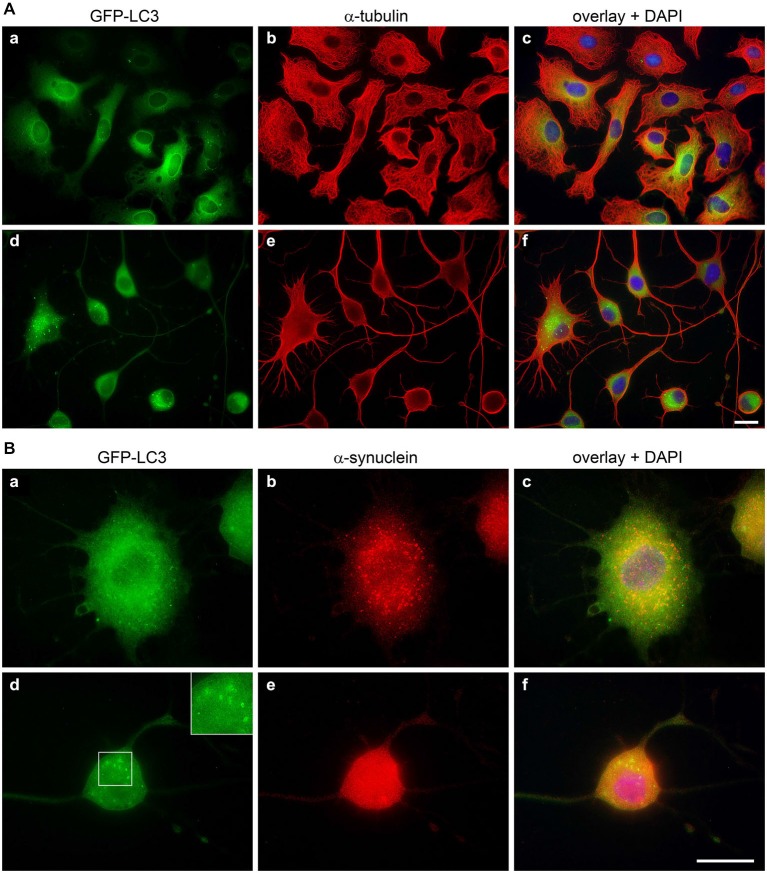
**Inhibition of UCH-L1 leads to the formation of GFP positive autophagic vesicles**. OLN-t40-α-syn-GFP-LC3 cells were incubated with 40 μM LDN for 18 h (d–f). Untreated control (a–c). **(A)** Cells were subjected to indirect immunofluorescence staining with an antibody against α-tubulin (red). GFP, green. Overlay with DAPI (blue). Scale bar: 20 μm. **(B)** Cells were subjected to indirect immunofluorescence staining with an antibody against α-syn (red). GFP, green. Overlay with DAPI (blue). The insert highlights the presence of GFP-positive autophagic vesicles after LDN treatment. Scale bar: 20 μm.

**Figure 9:**
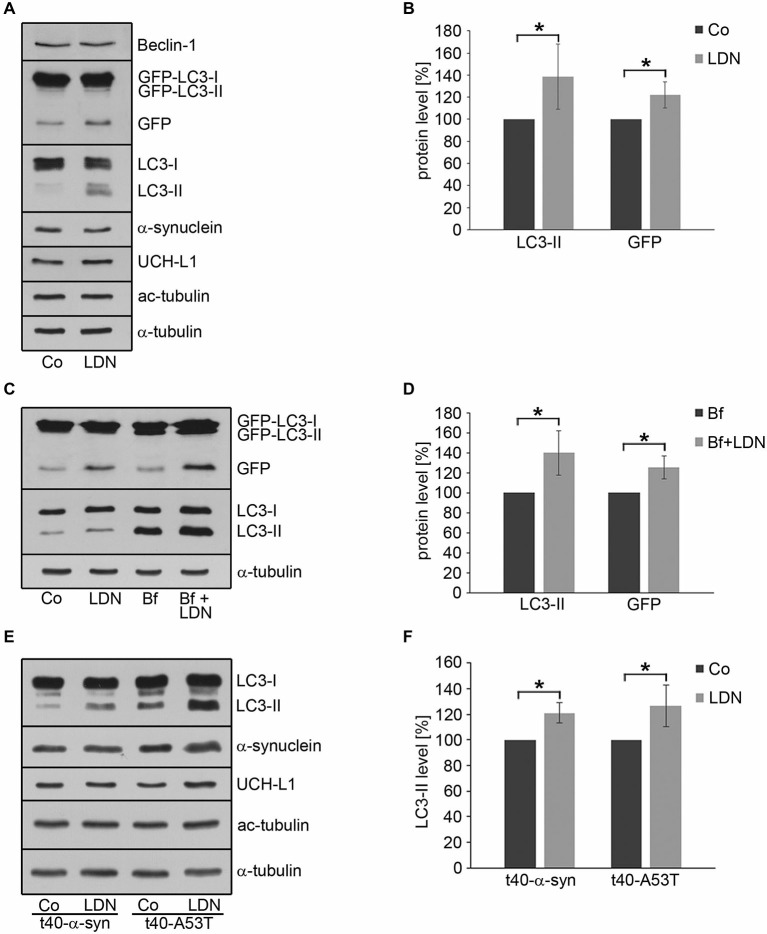
**Inhibition of UCH-L1 in oligodendroglial cells induces autophagy. (A)** OLN-t40-α-syn-GFP-LC3 cells were incubated with 40 μM LDN for 18 h (Co, untreated control). Cell lysates were subjected to immunoblot analysis with the antibodies as indicated on the right. **(B)** Quantitative evaluation of the blot shown in **(A)** was carried out using ImageQuant software. Data represent the mean ± SD from three independent experiments. LC3-II and GFP levels were normalized to α-tubulin and the total amount of the control was set to 100%. Statistical evaluation was carried out by Student’s *t*-test: *p < 0.05 significant. **(C)** OLN-t40-α-syn-GFP-LC3 cells were left untreated (Co), incubated with 40 μM LDN for 18 h, incubated with 3 nM bafilomycin A1 (Bf) for 20 h, or incubated with 3 nM Bf for 2 h followed by 40 μM LDN for 18 h. Cell lysates were subjected to immunoblot analysis with the antibodies as indicated on the right. **(D)** Quantitative evaluation of the blot shown in **(C)** was carried out as described above. LC3-II and GFP levels were normalized to α-tubulin and the total amount of the Bf treated cells were set to 100%. **(E)** OLN-t40-α-syn cells and OLN-t40-A53T cells were incubated with 40 μM LDN for 18 h (Co, untreated control). Cell lysates were subjected to immunoblot analysis with the antibodies as indicated on the right. **(F)** Quantitative evaluation of the blot shown in **(E)** was carried out as described above. LC3-II levels were normalized to α-tubulin and the total amount of the control was set to 100%.

In OLN-t40-α-syn cells under control conditions small α-syn positive aggregates were formed, which were not visible after treatment with LDN (Figure 5). To assess whether this was due to the activation of the autophagic pathway, we established OLN-t40-α-syn cells stably transfected with GFP-LC3 (MT-associated protein 1 light chain 3). These cells represent a suitable model system to assess the amount of GFP-LC3 puncta by immunofluorescence and to detect the GFP fragments generated by GFP-LC3 inside the autolysosome by immunoblot procedure using an anti-GFP antibody (Mizushima et al., 2010; Klionsky et al., 2012). During autophagosome formation endogenous LC3 is processed to LC3-I, an 18 kDa cytosolic isoform, which is converted to LC3-II, migrating at 16 kDa. LC3-II is tightly associated with the autophagosomal membranes and serves as a marker for autophagosomes. Its amount when normalized to tubulin or actin correlates with the number of autophagosomes. Figure 8 demonstrates that after treatment with LDN cells depict an increased level of GFP-LC3 puncta and similarly to OLN-t40-α-syn cells cellular processes are elongated and extremely thin. Furthermore, α-syn aggregates are removed after LDN treatment (Figure 8B). Next we addressed the question whether LDN activates the autophagic pathway and the autophagic flux, which comprises the dynamic process of autophagosome formation and the delivery to and degradation of the substrates within the lysosomes. Immunoblot analysis corroborates that after treatment with LDN the levels of LC3-II and free GFP are significantly increased, indicating that the autophagic flux was activated (Figures 9A,B). The level of Beclin-1, which is involved in the formation of the pre-autophagosomal membrane, did not change. To further sustain that the autophagic flux is not impaired, cells were preincubated with the lysosomal inhibitor bafilomycin A1 (3 nM) alone for 20 h, or for 2 h followed by LDN treatment (18 h) in the presence of bafilomycin A1. Immunoblot analysis shows that after the combined treatment the levels of LC3-II and GFP are significantly higher than after each compound alone, which indicates that the autophagic flux was activated (Figures 9C,D). An increase in LC3-II was similarly observed in oligodendroglial cells not expressing the GFP-LC3 fusion protein, i.e., OLN-t40-α-syn cells and of OLN-t40-A53T cells (Figures 9E,F). Hence, LDN activates the autophagic pathway and prevents the formation of α-syn aggregates in oligodendroglial cells.

## Discussion

UCH-L1 has been suggested to be important for neuronal health and synaptic stability, and to maintain a sufficient level of mono-ubiquitin, which is important for synaptic integrity (Todi and Paulson, 2011). UCH-L1 colocalizes with α-syn in presynaptic terminals, both proteins interact and have been implicated in PD (Lowe et al., 1990; Amerik and Hochstrasser, 2004; Yasuda et al., 2009). Here we show that UCH-L1 is also a constituent of ODC, the myelin forming cells of the CNS, and can associate with GCIs in the brains of patients with MSA. Its presence in GCIs further adds to the knowledge that dysfunctions of the proteolytic degradation systems and of the ubiquitin dependent pathways may contribute to intracellular inclusion body formation and neurodegenerative diseases. Furthermore, our data, using the specific inhibitor LDN demonstrate that UCH-L1 is critically involved in maintaining the cellular architecture in particular by influencing the MT network, and that its inhibition has an impact on α-syn aggregate formation and activates the autophagic pathway.

UCH-L1 inhibition in oligodendroglial cells led to the formation of elongated cellular processes, which were characterized by the presence of thick bundles of MTs positively stained by antibodies against acetylated and detyrosinated α-tubulin. Post-translational modifications of tubulin are critically involved in MT regulation and stability, process outgrowth and establishment of cell morphology and maturation. Tubulin acetylation is considered to occur on stable MTs and may selectively stimulate intracellular dynamics, and enhance intracellular vesicular traffic and cargo distribution (Janke and Bulinski, 2011; Perdiz et al., 2011). In oligodendroglia control cells ac-tubulin is evenly distributed within the soma and the cellular processes, however, as demonstrated by high resolution STED microscopy, interrupted by small gaps. In contrast thereto, after inhibition of UCH-L1 ac-tubulin is hardly seen in the soma but prominently expressed in the long cell processes. Here acetylated MTs are enriched and appeared to be bundled in the cellular extensions and are continuously immunostained without interruptions. Similarly, detyrosinated dense bundles of MTs are abundant within the cellular processes after LDN treatment, while in control cells immunostaining with antibodies against detyrosinated α-tubulin was detected mainly in the cell soma and not in the cellular extensions or edges of the cells. Detyrosination of α-tubulin has been linked to MT stability. However, detyrosinated MTs are not stabilized by detyrosination of α-tubulin *per se*, but this posttranslational modification indirectly stabilizes MTs, because they are less susceptible and protected from depolymerization (Fukushima et al., 2009; Janke and Bulinski, 2011). Hence, blocking UCH-L1 has a direct impact on the stability of the MT network by affecting tubulin posttranslational modifications, i.e., acetylation and tyrosination.

We were not able to detect ubiquitinated tubulin in our system, which also may be due to a very low level of ubiquitination. Only little is known about a possible role of tubulin ubiquitination. The ubiquitin ligase parkin was suggested to enhance the ubiquitination and degradation of misfolded α- and β-tubulin (Ren et al., 2003). Bheda et al. (2010) showed that UCH-L1 decreased the ability of tubulin to form MTs *in vitro*, and overexpression of UCH-L1 in an SV40 transformed fibroblastic cell line caused the reduction in MT assembly in about 30% of transfected cells. Yet, the question remained whether α- and β-tubulin were direct targets of ubiquitination (Bheda et al., 2010). Low molecular weight MAPs could also be involved in UCH-L1 mediated inhibition of tubulin polymerization. However, we did not observe differences in the action of LDN in the presence or absence of tau.

UCH-L1 binds to small polyubiquitinated proteins and cleaves ubiquitin molecules, thus maintaining a stable mono-ubiquitin pool, which is used for ubiquitination reactions (Todi and Paulson, 2011; Ristic et al., 2014). Besides its hydrolase activity, UCH-L1 can act as an E3 ligase responsible for K63-linked polyubiquitination of α-syn, which may block its proteasomal degradation (Liu et al., 2002). However, this requires dimerization of UCH-L1 and so far has only been seen *in vitro*. Ardley et al. (2004) described that after overexpression of UCH-L1 in cultured neuronal and non-neuronal cell lines, aggresomes were formed after proteasomal inhibition, which were surrounded by an MT cage-like structure, and besides UCH-L1 contained parkin and α-syn. This further sustains the notion that UCH-L1 plays a pathological role in inclusion formation, e.g., in PD, and may modulate the turnover of α-syn. Membrane associated UCH-L1 has been reported to promote α-syn neurotoxicity by possibly negatively regulating the lysosomal degradation of α-syn (Liu et al., 2009). Furthermore, under pathological conditions, when α-syn was overexpressed, blocking UCH-L1 by LDN resulted in an increase in LC3-II, which was not observed in control cells, suggesting a differential role of UCH-L1 activity under normal and pathological conditions (Cartier et al., 2012). In oligodendroglial OLN cells on the other hand, inhibition of UCH-L1 caused the induction of the autophagic pathway even in the absence of α-syn.

Prefibrillary oligomers of α-syn have been hypothesized to provide intermediates of fibrillary aggregates and inclusion bodies, and to constitute the toxic species causing neurodegeneration (Lansbury and Lashuel, 2006; Jellinger, 2009; Steiner et al., 2011; Kalia et al., 2013). α-Syn oligomers can be degraded by lysosomal pathways (Lee et al., 2004), and stimulating these pathways may be an effective therapeutic approach to prevent α-syn accumulation and toxicity. In the present cell culture system, the stable expression of α-syn leads to small α-syn aggregates, which do not stain with thioflavine S and represent non-fibrillar inclusions, which might precede the formation of fibrillary deposits (Riedel et al., 2009). Pharmacological inhibition of UCH-L1 attenuated the formation of these aggregates by activating the lysosomal pathway. Using GFP-LC3 expressing cells, we demonstrate that LDN increased the level of LC3-positive autophagic vesicles and of free GFP. Free GFP is generated by the degradation of GFP-LC3 within the autolysosomes indicating that the autophagic flux is activated. However, the total level of α-syn did not change, which points to the conclusion that stimulation of the autophagic flux effectively balanced the equilibrium of newly synthesized α-syn and its degradation, which contributes to the clearance of small oligomeric aggregates.

In summary, UCH-L1 is present in ODC and a constituent of GCIs in MSA brains. Inhibition of UCH-L1 causes the upregulation of autophagy, which is involved in the removal of α-syn aggregates. Furthermore, UCH-L1 inhibition caused the recruitment of detyrosinated and acetylated MTs to the cellular processes, which promotes their stabilization and possibly their association with dynein and kinesin motor proteins. This might sustain transport processes required to efficiently deliver proteins to the autophagic machinery. DUBs are central players in the regulation of protein ubiquitination and the UPS and thus crucial in many cellular regulatory mechanisms. Our data add to the knowledge that the UPS and the autophagy-lysosomal pathway are tightly balanced. DUB inhibitors provide a useful tool to investigate the manifold functions of DUBs in nerve cells and glia and to elucidate their dysregulation in neurodegenerative diseases.

## Authors Contributions

CR, KP, conceived and designed experiments. KP, performed the experiments. CR, KP, analyzed the data. CR, contributed reagents/materials/analysis tools. CR, KP, wrote the paper.

## Conflict of Interest Statement

The authors declare that the research was conducted in the absence of any commercial or financial relationships that could be construed as a potential conflict of interest.
